# Self-Regulation for and of Learning: Student Insights for Online Success in a Bachelor of Nursing Program in Regional Australia

**DOI:** 10.3390/nursrep11020035

**Published:** 2021-05-20

**Authors:** Blake Peck, Andrew Smith, Daniel Terry, Joanne E. Porter

**Affiliations:** School of Health, Federation University, P.O. Box 663, Mt. Helen, Ballarat, VIC 3350, Australia; b.peck@federation.edu.au (B.P.); d.terry@federation.edu.au (D.T.); joanne.porter@federation.edu.au (J.E.P.)

**Keywords:** online learning, nursing education, blended online learning

## Abstract

The blended online digital (BOLD) approach to teaching is popular within many universities. Despite this popularity, our understanding of the experiences of students making the transition to online learning is limited, specifically an examination of those elements associated with success. The aim of this study is to explore the experiences of students transitioning from a traditional mode of delivery to a more online approach in an inaugural BOLD Bachelor of Nursing program at a regional multi-campus institution in Victoria, Australia. Fifteen students across two regional campuses participated in one of four focus groups. This qualitative exploration of students’ experience contributes to contemporary insights into how we might begin to develop programs of study that help students develop self-regulation. A modified method of thematic analysis of phenomenological data was employed to analyse the focus group interview data to identify themes that represent the meaning of the transition experience for students. This qualitative exploration of students’ experience contributes to contemporary insights into how we might begin to develop programs of study that help students develop self-regulation.

## 1. Introduction

Offering an online platform for learning in Higher Education has provided an opportunity to expand the geographic footprint of education provision. Currently, the blended approach to teaching and learning is utilised in the higher education sector as a vehicle to promote effective and enhanced learning experiences for students. The move to online learning is growing rapidly as a means of delivering learning experiences to students off and on campus [1,2]. Online learning has experienced such growth that an exploration of teaching excellence without due consideration to online strategies either on their own or in combination with other traditional methods would be entirely unreasonable [3,4]. Despite increased flexibility in teaching and learning experience, as well as the potential for financial savings for higher education institutions [3,5,6], there are concerns about the effectiveness of using technology to improve the student experience [7].

A more recent approach has been a blended pedagogy for learning. The blended approach combines didactic teaching methods with learning activities enhanced or supported by technology [3]. This approach provides the conduit for online learning into programs that have traditionally adopted didactic pedagogy. In health professional education, students are required to have a combination of learning experiences in both clinical skills and theoretical principles and their application in clinical practice. In combination, this means students in health professional programs of study will continue to experience increasing levels of online learning within the blended approaches adopted [2].

Means et al. [8], reporting on a meta-analysis exploring the impact on learning for students across traditional, blended and online learning platform reported that students in the online and blended cohorts performed better with improved learning outcomes when compared to others. This is echoed by Berga et al. [2], who reported specifically on increased learning outcome measures amongst those students who engaged in blended learning when compared to online only. Bernard et al. [9], suggests that differences such as these are more reflective of the type of learner who chooses to study in these modes, more so than the impact of the mode itself. That is, a student who chooses to learn online may well be more inclined to flexibility and independence of learning while a student enrolled in a blended mode of delivery might prefer structure and the social aspects of learning that come from a blended mode.

If the assertion above is correct, then one constant characteristic of student experience will be a reduction in structured class time, with less direct contact with facilitators and subsequently a greater reliance upon self-regulated strategies for learning [2,10]. There is an unequivocal and unavoidable increase in the level of self-regulated learning that we see across the higher education sector at present, in conjunction with a growing body of work that establishes the links between greater levels of self-regulation in academic performance [11]. Despite the importance of learning preferences and their links to learning outcomes in the context of increasing use of online learning modalities in health education, there is a lack of research exploring the strategies that students use to navigate their experiences of learning in an online or blended mode of study [2,3,4]. More specifically, there is a lack of qualitative research that explores the ‘lived experience’ of students in an online or blended mode of delivery [3]. This article seeks to give voice to students enrolled in a health-related program of study by focusing on their experiences following transition from a traditional face-to-face mode of learning to an online blended approach finding ways to learn.

This study contributes to the discussion of the blended and online learning approach being adopted in higher education globally, particularly within health-related programs of study. This study seeks to provide insights into the experiences of students engaged in the transition from face-to-face learning to online learning. While we take an Australian context as an exemplar, we have sought to extract data at a particularly high level of theoretical abstraction so as to provide a valuable touchstone for other educators seeking to also make the transition to a blended and online learning platform in ways that are transferable to other disciplines of study.

## 2. Materials and Methods

### 2.1. Research Design

A hermeneutic phenomenological approach informed by the philosophy of Hans-Georg Gadamer was utilised to underpin the qualitative component of this study. The ontological nature of the philosophy enabled an exploration of the everyday meanings that students constructed of their experiences with an of the transition to an online learning approach [12,13]. Moreover, the hermeneutic phenomenological approach enabled the essence of the experience to be explored through the words of those people immersed within the phenomena itself.

### 2.2. Setting

This study focusses on an Australian context in a regional University in Australia with multiple campuses. Bachelor of Nursing programs in Australia have a registration requirement for students to undertake psychomotor clinical laboratory learning session as part of their learning which requires students to be in a simulated environment for learning, making the development of a completely online nursing program problematic. Learning therefore mandates that students attend some face-to-face sessions. The nursing program in this study is delivered via two methods, a *standard* face-to-face delivery with students engaging with online interactive content attending weekly face-to-face active learning sessions on campus. The *flexible* delivery model involves content and interactivity exclusively using an online medium, with a residential face-to-face learning block once per semester for clinical skills sessions. Within this program, areas of learning are grouped together to form modules of learning, and a module includes the pre-reading, the lecture, the tutorials and any additional extension components for immersive learning.

### 2.3. Participants

Participants in this study included those who were already enrolled in the program and had made the transition from a traditional mode of face-to-face weekly delivery to an online (or flexible) platform the following academic year (*n* = 9), and those students who entered the program for the first time in a flexible mode (*n* = 6), with no experience in the traditional delivery mode. Participants were students from across two regional campuses of the university. Fifteen participants engaged in this study, with the vast majority female, over 25 years of age, married, employed, and having children.

### 2.4. Data Collection

Student focus groups were conducted by two senior academics using a semi-structured interview technique. Four focus group interviews were conducted. Focus groups were audio-recorded and underwent verbatim transcription for analysis. A question schedule was created to ensure a degree of consistency. Students were questioned in an effort to generate free dialogue: ‘Can you tell me about your experiences of being a student in the new curriculum?’ In the event that the conversation slowed down, further focused questions were initiated.

### 2.5. Data Analysis

A modified version of the technique for phenomenological data analysis (verbatim transcription, extraction of significant statements, identify similarities in formulated meanings, group the similar meanings, create an exhaustive statement) proposed by Collaizi [14], was used for thematic development in order to uncover the hidden and ambiguous nature of the experiences of the student/stakeholders interviewed. In-depth contact with the transcripts and the audio-recordings was maintained and supported the identification of significant statements that pertained to the phenomena of interest. The meanings formulated were then clustered together to form themes that encompassed the experiences of students. The researchers conducting the interviews worked with other members of the team to ensure accuracy of the data for analysis through comparing the transcripts with the audio-recordings to ensure that rigour was established. Where discrepancies arose in the interpretation of the data, a discussion amongst the entire research team was had to reach consensus.

## 3. Results

Students described the experience of moving from a face-to-face to an online format as presenting a newfound freedom to develop their own strategies to learn. Students recognised that their previous face-to-face timetable provided a degree of structure and students were faced with finding ways to manage their own time, space and learning. The overwhelming theme to emerge was one of ‘self-regulation’. This theme embodies finding new ways to navigate the tension between study, clinical practice, family, employment, and life. The theme, self-regulation, is expanded by way of three subthemes: ‘*Tailoring learning with life*’, ‘*Strategies for success—the meat and potatoes*’, and *‘Titrating learning’*. In combination, these themes come to represent and build the context of those participants making, or having made, the transition to an online learning environment in a Bachelor of Nursing program.

### 3.1. Self-Regulation

The major theme, self-regulation, speaks largely to a transition from a state of upheaval to one of learning success. The theme can be considered a spectrum with two ends which captures best the experiences of students in this study. At one end of the spectrum, students describe a sense of needing to self-regulate for learning in order to then engage in self-regulation of learning at the other end of the spectrum. Self-regulation for learning embodies the initial upheaval described by students when they realised that their learning, and by association aspects of their life, were no longer organized, having lost the security of a ‘timetable’. The self-regulation for learning end of the spectrum identifies with the way in which students describe having to find new ways of being a student by navigating the immediacy and complexity of their own life, family, employment, etc., in order to first clear a pathway for learning. Importantly, students did not describe these preparatory stages in terms of a mere ‘checklist’ of items to be dispatched; instead, they describe this as a shift in their own way of being in order to outsource or resource aspects of their traditional roles.

Once the preparatory groundwork had been laid, students describe being able to then reach a state where they recognised the self-regulation of learning as an experience that gave them a tremendous sense of control over their learning. With the majority of respondents having experience with the traditional model of teaching and having transitioned to a flexible or online mode, students were in a position to reflect on their new-found capacity to self-regulate their learning with authority. Students recognised that being able to choose the time, pace and space in which they learnt allowed for a greater sense of emersion in learning materials. This end of the self-regulation spectrum that is embodied by this theme aligned with key learnings for success in an online program. Immersed in the self-regulation of their learning students describe being able to capitalise on opportunities for learning success such as being better able to concentrate on their learning.

While the theme of self-regulation can be seen across the experiences of all respondents, it is the word of one respondent that captures the essence of the major theme of self-regulation embodying aspects of both self-regulation for and self-regulation of learning:

*For me, I’ve never liked the classroom side of things because half the time you’d fall asleep in class. Whereas being online, I could do it when I was ready. It’s all about time management.*

The supporting subthemes provide an insight into the major theme and emphasise the nuances of the student’s experience as they transition to a new mode of learning. These subthemes include ‘Tailoring’ Learning to Life; Strategies for Success, and Titrating Learning.

### 3.2. Tailoring’ Learning with Life

Tailoring learning with life refers to the ‘self-regulation for learning’ end of the spectrum and focuses on the adaptations that participants underwent in attempting to balance learning with their everyday lives. Participants pointed strongly to tailoring and balancing their lives around study, work and family commitments across all hours of their day. This appeared to become a release for students from being tethered to the weekly routine of attending lectures and classes on campus. Students who chose to study in a flexible mode (online with residential blocks) highlighted their newfound ability to self-regulate as a positive characteristic of their learning as it allowed them to ‘choose’ or tailor when and what to do in terms of study, work and family.

Tailoring learning with life also brings to light the continuity that exists between self-regulation for and self-regulation of learning. We suggest that these ideas represent the opposite poles of the same theme, and here we see the relationship between the two poles. Students who had engaged in self-regulation for learning were able to capitalise on the gains made and engage in self-regulation of learning by choosing where and when they direct their attention to achieve the greatest gains. For instance, some participants made reference to the convenience of being able to ‘choose to study or choose to work’. One participant highlights:

*[It] was good knowing, ‘oh I have a shift coming up tonight, but I have two modules worth of work to do. But I can do this when I get home.’ It’s not, ‘I can’t work because I’ve got a lecture at Uni (sic).’*

Similarly, another participant highlights the benefits of being able to engage in self-regulation of learning, having been successful in self-regulation for learning:

*I didn’t have a particular time in the day when I’d do the module work, but what I did find handy was that I could concentrate on things that were a bit more important.*

These responses highlight the convenience of being able to focus on study and engage with content and learning at the most crucial times. It also brings to light that learning is a journey that is different for each student and their competing priorities are diverse. Self-regulation for and self-regulation of learning involves the tailoring of study for ‘best fit’ with work commitments and family.

Participants provide some insight into the concept of engagement suggesting that self-regulation for learning to be negotiated or navigated to provide maximum opportunity in engaging the self-regulation of learning end of the spectrum. Participants describe a high degree of discipline in their approach to learning through self-discipline, time management and commitment to learning supporting self-regulation of learning. Participants identify the ability to take more control and to commit to a self-managed schedule as paramount in a flexible mode in order to yield outcomes. This is particularly evident through responses of participants:

*I had to take more control of my learning and I think that helped me learn more*

*I am rather strict with my routine, but I do have flexibility. So I went flex (sic). The best thing I ever did.*

In this subtheme, students adapt and modify their lives to include those they perceive to be most important at any given point in time. This provides a distinct contrast to traditional methods of learning in that students were dictated to by institutional regulations of timetables and expectations of attendance, which requires students to shift their focus and their lives This subtheme highlights that the self-regulation of learning involves a complex tailoring with one’s own life that is only possible if one has successfully self-regulated for a pathway to learning.

### 3.3. Strategies for Success

Arguably the most central feature of self-regulation of learning is the ability of students to engage with material so that their learning is deeper. Having self-regulated for learning and moving to a self-regulation of learning, students described some strategies that they use for learning. One of the most central success strategies that the students identified in the careful balance between life and learning was that around being selective of what they needed to learn. Students describe finding strategies to focus on the more important aspects in order to prioritise elements of learning. The following quote from a participant captures the responses embodied by others a potential for students to able to select aspects from content that is most important and to do the learning associated with that:

*I would go through the module and it’s like okay, that’s really important, do the activity, and write my notes down in my book and on my papers.*

Similarly, other respondents referred to an approach where they choose to engage in the content or activities that they perceived to be of the most importance. One respondent described:

*[The lecturer] would go this is the meat and this is the potatoes, and you would get your highlighter out, okay this is what we need to know.*

This theme helps to describe a process in which students engage in reviewing online content to determine which components were considered more critical to learning, vital for assessment, and what elements were considered the ‘extras’. One participant in this study also referred to this strategy as the *‘fluff’*. In the process of developing strategies for success, self-regulated learners were more likely to focus their learning based on what they needed to know (the meat and the potatoes) and what might be nice to know, and what is the ‘fluff’.

As proposed within this subtheme, students speak to the value of self-regulation of learning by being able to identify and focus on the larger more salient aspects of learning the more important (the meat) whilst also being able to discern the additional elements (the potatoes). To an extent, students pointed to a process in which they identified things that they needed to know and would be nice to know. There is also some suggestion within this theme that students who had previously engaged in the traditional method of teaching had translated this approach into the flexible learning space. Cognizant that the onus of discerning the *meat* from the *potatoes* seems to have shifted towards the student in the new mode of delivery, it could be suggested that this subtheme highlights the ‘translation’ of traditional learning practices into the flexible space, adding further support to the self-regulation of learning theme.

### 3.4. Titrating Learning

While the major theme and subthemes have been described with self-regulation in mind, a number of participant responses point to a process in which they select, immerse, and binge learn as a strategy for success. This self-regulation of learning strategy is reminiscent of the process of titration, where adjustments are made according to competing or immediate demands (e.g., work, life, and study) which is a mechanism to support self-regulation for learning. Here, again, we can see the interplay between the two ends of the same spectrum. Much like a see-saw, a student makes subtle shifts in self-regulation for and self-regulation of learning to help balance demands:

*You have] the freedom to do it at your own pace. Like of course, there was a due date, but you could do it when you were ready. There’s a lot more freedom, and a lot less time restrictions”*

In addition, another respondent added further contextual elements that support the titration of learning with life:

*I do my afternoon activities, which possibly could be dinner, cleaning, footy (football). They (the children) and then I jump back on Moodle (online learning management system) and finish off before I go to sleep”.*

The emphasis that students placed around the awareness of flexibility and the reported ability to engage and immerse themselves in learning at their own pace, in their own time, feeds strongly into the major theme of self-regulation of learning. It could be proposed that the ability for students to clearly identify that they were in control of the self-regulation of and for learning using strategies for success (“the meat and potatoes”) could produce and empower them to be successful. These subthemes illuminate the importance for nursing curriculum to support students to be self-regulated and therefore in control of their own learning.

## 4. Discussion

The findings draw parallels with the literature relevant to self-regulated learners and to the self-efficacy benefits of online learning reported by others [2,10,15]. Strategies of self-regulation such as organisation, goal setting and planning seem to feature strongly in the student’s responses and are highlighted by current research. Self-regulation in learners is described as an ability to be self-motivated or proactively engaged in the organisation and planning of their learning [2,10]. Berga et al. [2] and Artino and Jones [16], posit that students studying in an online mode tend to demonstrate much more control and self-efficacy over their study habits and success if they perceive the learning environment to be a positive emotional experience. Clearly, these components underpin the themes identified in this current study and it is evident that students studying in an online environment were able to identify a self-regulation for learning. This study has identified the major theme of self-regulation amongst nursing students, and a series of subthemes that point to strategies that support their ability to make autonomous decisions around balancing life and learning in order to extract the most from their opportunities for learning through prioritisation. Many of the participants in this study focused on elements of self-regulation for learning and the ability to adapt as things in life provide additional challenges. We suggest that this could stem largely from the demographic of this current study, who are largely mature-aged, females working part or full time with family commitments.

Participants made clear that the two ends of the same spectrum, self-regulation for and self-regulation of learning, exist in a complex balance, much like a seesaw. Students recognised that they needed to shift the balance in favour of life at critical times and sacrifice aspects of learning to take on focus on only those elements that are more critical. This careful balance or titration of learning could be considered as reflective of findings from Berry and Hughes [17], who outline the benefits of online learning in which students claim to relate largely to logistical benefits that online learning offers. Compared to traditional methods of classroom-based and timetabled learning, the use of technology and flexible online learning removed the need for participants to have to travel and be present in class, thus enhancing autonomy and student satisfaction overall. It is clear from this study that the ability to titrate the self-regulation of and self-regulation for learning increased with the more positive and in control the participants felt. This speaks largely to the concepts of both autonomy and flexibility or self-regulation as Berry and Hughes [17], put forward. While representing different disciplines of learning as a means of contrast between the present study and existing literature, participants in this study did not seem to indicate feelings of isolation and disconnection as Berry and Hughes [17] outline; rather, students appeared to have built strong networks through their online learning management systems and the mode of delivery described.

While it was not necessarily the aim of the present study to explore the more cognitive elements of the transition to online learning, there is some degree of overlap that can be seen with other studies in this area. Broadbent [10], describes the processes of rehearsal, elaboration and critical thinking as central to self-regulation. The strategies for success, *‘the meat and potatoes’,* that participants in this study highlighted represent less about cognitive mechanisms and much more in terms of the type of learning that students engage with. Students in this study appear to be pragmatic learners, making decisions about learning and how they can best ‘titrate’ learning in terms of the rest of their lives to maintain a self-regulation for and of learning. Interestingly, Muir et al. [18], makes reference to the concept of interaction and levels of engagement with others in an online learning platform, i.e., lecturers, peers and online content, as elements to success. In contrast, students placed greater emphasis on the interaction with content. Reflecting the study by Berga et al. [2], little reference was placed on a reliance on their peers or others for their learning. This may be linked to the fact that a large proportion of participants had some life experience, being mature aged and having other significant outside commitments to attend to.

### 4.1. Limitations

The most evident limitation is the qualitative nature of this study. With the limited student numbers taking part, it is difficult to make significant claims about the transferability of the findings into other settings. Ongoing research on the experience of students who are undertaking an online program of study in a multitude of discipline areas would continue to establish a deeper understanding of the experience over a larger cohort, particularly taking careful consideration for age, socioeconomic and cultural background. A larger sample could also begin to address the question of differential effectiveness of online learning approaches across different disciplines of higher education.

### 4.2. Implications for Practice

The findings from this study provide a deeper understanding of the experiences for students who are transitioning to or are enrolled into an online mode of delivery. It is evident from this exploratory study that students experienced an initial sense of upheaval at having to find ways to manage their busy life in order to be prepared for an online learning. While our study identified that this initial feeling of being overwhelmed disappeared, it does highlight an important milestone that should be acknowledged by educators. We believe that having a period of more intense or frequent contact with students initially will provide the necessary structure that is first needed to achieve self-regulation.

## 5. Conclusions

This small-scale study undertaken in an Australian regional University with students who have migrated from face-to-face delivery to blended online flexible curriculum touches on ways students perceived the impacts on their learning in higher education. Participants described elements of their experience that we have represented by way of a central theme *self-regulation*. The experience of making the transition to a new mode of learning required that students developed skills of self-regulation for learning as well as self-regulation of learning. Central to the experience of those students within this study is the precarious balance of self-regulation for learning on the one hand and self-regulation of learning on the other. Here, students find ways to manage their existing role commitments to then engage in self-regulation of learning by identifying areas of most importance to their overall learning. These pragmatic decisions about learning are, to a large extent, determined by the degree with which students’ must titrate their learning in order to address the immediate issues that arise in their lives. Interestingly, in the initial recognition of a need for self-regulation for learning, students reported feeling that the newer approach to their learning presented as an imposition and therefore they found it hard to progress to a position of self-regulation of learning where the students capitalises on the convenience and flexibility that this balance created for incorporating learning into and around their own lives. The journey towards self-regulation is not simple for students but increasing our understanding of this helps guide educators to strategically incorporate tasks and learning that are perhaps smaller ‘chunks’ of learning and assessments that can be easily titrated into the lives of students navigating the complex balancing act of life and learning that confronts the contemporary student.

## Data Availability

The data presented in this study are available on request from the corresponding author.
